# A new genus and species of Hydrobiidae Stimpson, 1865 (Caenogastropoda, Truncatelloidea) from Peloponnese, Greece

**DOI:** 10.3897/zookeys.1037.64038

**Published:** 2021-05-18

**Authors:** Andrzej Falniowski, Jozef Grego, Aleksandra Rysiewska, Artur Osikowski, Sebastian Hofman

**Affiliations:** 1 Department of Malacology, Institute of Zoology and Biomedical Research, Jagiellonian University, ul. Gronostajowa 9, 30-387 Kraków, Poland Jagiellonian University Krakow Poland; 2 Horná Mičiná 219, SK-97401 Banská Bystrica, Slovakia Unaffiliated Banská Bystrica Slovakia; 3 Department of Animal Reproduction, Anatomy and Genomics, University of Agriculture in Krakow, al. Mickiewicza 24/28, 30-059 Kraków, Poland University of Agriculture Krakow Poland; 4 Department of Comparative Anatomy, Institute of Zoology and Biomedical Research, Jagiellonian University, ul. Gronostajowa 9, 30-387 Kraków, Poland Jagiellonian University Krak&oacute;w Poland

**Keywords:** Cytochrome oxidase subunit I, morphology, phylogeny, Pliocene flooding, speciation

## Abstract

Minute caenogastropod brackish-water gastropods, formerly classified as *Hydrobia*, are important elements of the brackish-water fauna and were objects of intensive study for many years. Until now, five genera have been distinguished, most of them represented by a number of species, but rather indistinguishable without molecular data (cytochrome oxidase subunit I – COI). In the eastern Mediterranean region, they are still poorly studied. In this paper, we present a new species of “*Hydrobia*” from the brackish Moustos spring, Arkadia, eastern Peloponnese, Greece. The shell, protoconch, radula, female reproductive organs, and penis are described and illustrated, together with the molecular (COI) relationships with other hydrobiids. All data confirm that these snails represent a distinct taxon, which must be classified as a new species belonging to a new genus. The formal descriptions are given. The closest, sister taxon is *Salenthydrobia* Wilke, 2003. The molecularly estimated time of divergence, 5.75 ± 0.49 Mya, coincides with 5.33 Mya, which is the time of the Oligocene flooding that terminated the Messinian salinity crisis. During the latter period, brackish “Lago-Mare” habitats were most probably suitable for the last common ancestor of *Salenthydrobia* and the newly described genus. Later, the Pliocene flooding isolated the Apennine and Peloponnese populations, promoting speciation.

## Introduction

The typically brackish-water caenogastropod snails formerly known as *Hydrobia* inhabit estuaries and other brackish habitats around the northern Atlantic and adjacent seas; in many places, these small, tiny snails are numerous and their biomass is large. Thus, they form an important component of the brackish water fauna and have been studied for many aspects (for details see Muus 1967; Falniowski 1987; Fretter and Graham 1994). The simple shells show a set of plesiomorphic character states and are extremely variable, making species determination hardly possible (e.g., Muus 1963, 1967; Falniowski et al. 1977; Wilke and Falniowski 2001). Muus (1963, 1967) demonstrated clear and stable differences in the penis morphology and snout and tentacle pigmentation between the three Baltic species, although the pigmentation was later found to be more variable but still useful for species determination (Falniowski 1986). However, later studies (e.g., Radoman 1973, 1977, 1983; Giusti and Pezzoli 1984; Wilke and Davis 2000; Wilke et al. 2000; Wilke and Pfenninger 2002; Wilke 2003), including those applying molecular data (mostly the partial sequences of mitochondrial cytochrome oxidase), confirmed these morphological differences, but as discriminating the genera. The discrimination of species within these genera was found possible only with molecular data. Currently, five genera are distinguished within *Hydrobia* s.l.

The genus *Hydrobia* Hartmann, 1821 (type species *Cyclostoma acutum* Draparnaud, 1805) is represented by several molecularly distinct species with rather restricted ranges, both Atlantic and Mediterranean. The nomenclatural problems (e.g., Bank et al. 1979; Falniowski and Szarowska 2002) were solved by the ICZN Opinion 2034 (Case 3087) (International Commission on Zoological Nomenclature 2003). The genus *Peringia* Paladilhe, 1874 (type species *Turbo ulvae* Pennant, 1777) is monotypic. *Peringia ulvae*, capable to live in salinity as high as at the open sea and having a lecithotrophic veliger, is widely distributed along the Atlantic coast, including the Baltic sea, but does not inhabit the Mediterranean Sea. The genus *Ventrosia* Radoman, 1977 (type species *Turbo ventrosus* Montagu, 1803) has been considered a junior synonym of *Ecrobia* Stimpson, 1865, since Davis et al. (1989) suggested that North American *Ecrobia truncata* (Vanatta, 1924) was introduced from Europe and would then be a synonym of *Ventrosia ventrosa*. This was later confirmed by molecular data (Osikowski et al. 2016). *Ecrobia* (its type species, following the ICZN rules, *Turbo ventrosus* Montagu, 1803) is represented by a few molecularly distinguishable species (Szarowska and Falniowski 2014; Osikowski et al. 2016) known from the Baltic Sea to the Black Sea. The genus *Adriohydrobia* Radoman, 1977 (type species *Paludina gagatinella* Küster, 1852) is known from the Adriatic Sea only; a few nominal species assigned earlier to *Adriohydrobia* are molecularly identical with *A. gagatinella*, and thus they became synonyms (Wilke and Falniowski 2001). The monotypic genus *Salenthydrobia* Wilke, 2003 (type species *Salenthydrobia ferreri* Wilke, 2003), was found at three closely situated populations in the southernmost part of the Apennine Peninsula (Wilke 2003).

Reliable data are scarce on the group formerly known as *Hydrobia* in the eastern Mediterranean brackish habitats. The numerous records that exist of *Peringia ulvae* from the eastern Mediterranean are a good example, as this species does not occur in the Mediterranean Sea. Another example is the monograph on the Greek Hydrobiidae (Schütt 1980), which does not mention any representatives of “*Hydrobia*”. Even though shells cannot be used for species determination, many hydrobiologists and marine biologists still record species determined by shell characters alone (e.g., Koutsoubas et al. 2000; Evagelopoulos et al. 2009). As stated above, the head pigmentation and penis morphology make identification of genera possible (Muus 1963, 1967; Falniowski 1986, 1987), while female reproductive organs are taxonomically less useful (Falniowski 1988). At species level, all morphological characters are hardly applicable because of morphostatic evolution (Davis 1992). Non-adaptive radiation, marked by the rapid proliferation of species without ecological differentiation (Gittenberger 1991), results in a flock of species that need not differ either morphologically or ecologically. Thus, at the species level in the Hydrobiinae, morphological characters cannot be used for species recognition alone, and molecular data are inevitably necessary to distinguish taxa.

So far, two species of *Ecrobia* have been recorded from six localities in Greece (Kevrekidis et al. 2005; Szarowska and Falniowski 2014; Osikowski et al. 2016), one of them (*E. ventrosa*) at the western coast of the Peloponnese Peninsula. In summer 2009, a few specimens of “*Hydrobia*” were collected at the brackish Moustos spring at Arkadia, on the eastern coast of the Peloponnese. The aim of the present paper is to establish their phylogenetic position, applying morphological and molecular data.

## Material and methods

**Figure 1.**
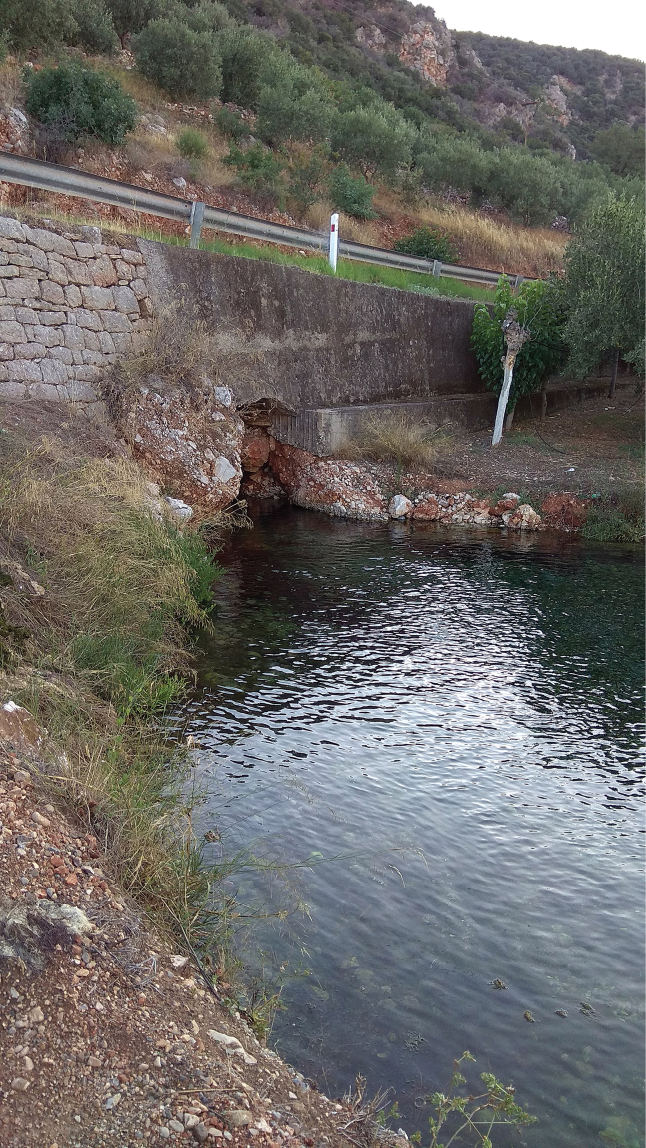
Moustos spring, type locality of *Achaiohydrobia moreana*.

The snails were collected in 2009 by sieve at the Moustos spring (Fig. 1), Arkadia, eastern Peloponnese, Greece (2 km N of Aghios Andreas, under the road from Astros to Korakovouni, 37.3845, 22.7444). The spring is situated about 500 m from the Aegean Sea. This large, brackish spring with sulphide content, rising from calcareous breccia, feeds a larger seashore lagoon called Limni Moustos with adjacent swamps hosting a bird reserve. The specimens were taken from the spring and the stony ridge towards the lagoon. No specimens were found in the lagoon.

The snails were fixed in 80% ethanol. The shells were photographed with a Canon EOS 50D digital camera, under a Nikon SMZ18 microscope with a dark field. The dissections were done under a Nikon SMZ18 microscope with a dark field, equipped with Nikon DS-5 digital camera, whose captured images were used to draw anatomical structures with a graphic tablet. Morphometric parameters of the shell were measured by one person using a Nikon DS-5 digital camera and ImageJ image analysis software (Rueden et al. 2017). The penes were photographed under Motic microscope with a dark field. The radulae were extracted with Clorox, applying the techniques described by Falniowski (1990), and examined and photographed using a HITACHI S-4700 scanning electron microscope.

Snails for molecular analysis were fixed in 80% ethanol. DNA was extracted from whole specimens; tissues were hydrated in TE buffer (3 × 10 min); then, total genomic DNA was extracted with the SHERLOCK extraction kit (A&A Biotechnology), and the final product was dissolved in 20 μl of tris-EDTA (TE) buffer. The extracted DNA was stored at −80 °C at the Department of Malacology, Institute of Zoology and Biomedical Research, Jagiellonian University in Kraków (Poland).

Mitochondrial cytochrome oxidase subunit I (COI) and nuclear histone 3 (H3) loci were sequenced. Details of PCR conditions, primers used, and sequencing were given by Szarowska et al. (2016a). Sequences were initially aligned in the MUSCLE (Edgar 2004) program in MEGA 7 (Kumar et al. 2016) and then checked in BIOEDIT 7.1.3.0 (Hall 1999). Uncorrected *p*-distances were calculated in MEGA 7. The estimation of the proportion of invariant sites and the saturation test (Xia 2000; Xia et al. 2003) were performed using DAMBE (Xia 2013). In the phylogenetic analysis additional sequences from GenBank were used as reference (Table 1). The data were analysed using approaches based on Bayesian inference (BI) and maximum likelihood (ML). We applied the GTR model whose parameters were estimated by RaxML (Stamatakis 2014). The Bayesian analyses were run using MrBayes v. 3.2.3 (Ronquist et al. 2012) with defaults of most priors. Two simultaneous analyses were performed, each with 10,000,000 generations, with one cold chain and three heated chains, starting from random trees and sampling the trees every 1,000 generations. The first 25% of the trees were discarded as burn-in. The analyses were summarised as a 50% majority-rule tree. The ML analysis was conducted in RAxML v. 8.2.12 (Stamatakis 2014) using the RAxML-HPC v. 8 on XSEDE (8.2.12) tool via the CIPRES Science Gateway (Miller et al. 2010). To calibrate the molecular clock for COI, the divergence time between *Peringia ulvae* and *Salenthydrobia ferrerii* (Wilke 2003), with correction according to Falniowski et al. (2008), were used. The likelihoods for trees with and without the molecular clock assumption in a likelihood ratio test (LRT) (Nei and Kumar 2000) were calculated with PAUP. The relative rate test (RRT) (Tajima 1993) was performed in MEGA. As Tajima’s RRTs and the LRT test rejected an equal evolutionary rate throughout the tree, time estimates were calculated using a penalized likelihood method (Sanderson 2002) in r8s v. 1.7 for Linux (Sanderson 2003).

**Table 1.** Taxa used for phylogenetic analyses with their GenBank accession numbers and references.

Species	COI/H3 GB numbers	References
*Adriohydrobia gagatinella* (Küster, 1852)	AF317857/-	Wilke and Falniowski 2001
*Belgrandiella kuesteri* (Boeters, 1970)	MG551325/MG551366	Osikowski et al. 2018
*Bythinella cretensis* Schütt, 1980	KT353689/-	Szarowska et al. 2016b
*Ecrobia grimmi* (Clessin in W. Dybowski, 1887)	MN167716/-	Vandendorpe et al. 2019
*Ecrobia maritima* (Milaschewitsch, 1916)	KX355830, KX355834/MG551322	Osikowski et al. 2016/Grego et al. 2017
*Ecrobia spalatiana* (Radoman, 1973)	MN167737/-	Vandendorpe et al. 2019
*Ecrobia truncata* (Vanatta, 1924)	MN167740, MN167741/-	Vandendorpe et al. 2019
*Ecrobia ventrosa* (Montagu, 1803)	KX355837, KX355840/-	Osikowski et al. 2016
*Hydrobia acuta* (Draparnaud, 1805)	AF278808/-	Wilke et al. 2000
*Hydrobia acuta neglecta* Muus, 1963	AF278820/-	Wilke et al. 2000
*Hydrobia glyca* (Servain, 1880)	AF278798/-	Wilke et al. 2000
*Littorina littorea* (Linnaeus, 1758)	KF644330/KP113574	Layton et al. 2014/Neretina 2014, unpublished
*Montenegrospeum bogici* (Pešić & Glöer, 2012)	KM875510/MG880218	Falniowski et al. 2014/Grego et al. 2018
*Peringia ulvae* (Pennant, 1777)	AF118292, AF118302/-	Wilke and Davis 2000
*Pontobelgrandiella* sp.	KU497012/MG551321	Rysiewska et al. 2016/Grego et al. 2017
*Pseudamnicola pieperi* Schütt, 1980	KT710670/ KT710741	Szarowska et al. 2016a
*Pseudorientalia* sp.	KJ920477/-	Szarowska et al. 2014
*Salenthydrobia ferrerii* Wilke, 2003	AF449201, AF449213/-	Wilke 2003

### Abbreviations

**GNHM**Goulandris Natural History Museum. Athens, Greece;

**HNHM**Hungarian Natural History Museum, Budapest;

**NHMW**Natural History Museum Vienna, Austria;

**NHMUK**The Natural History Museum, London UK.

## Results

**Figure 2.**
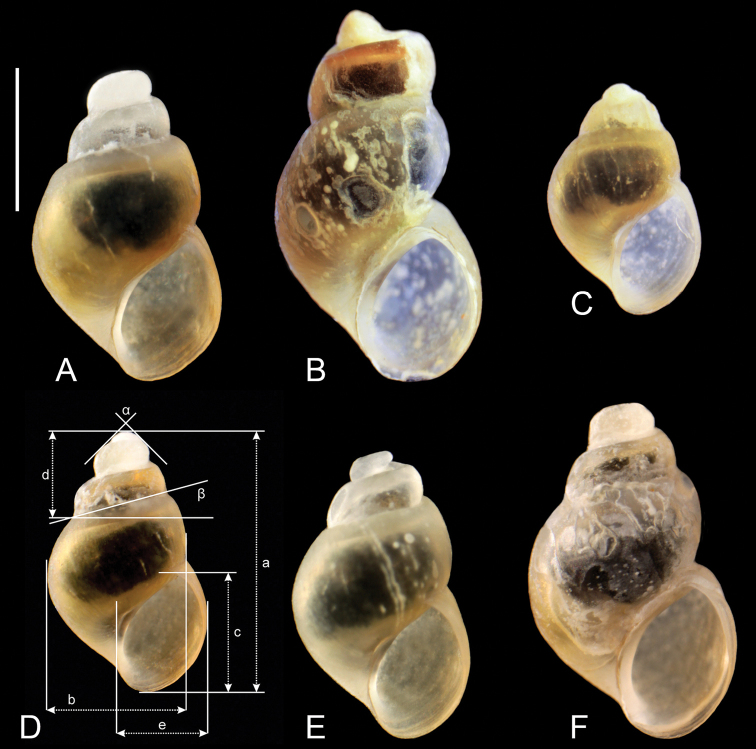
Shells of *Achaiohydrobia moreana***A** holotype; shell measurements are shown: a = shell height, b = body whorl breadth, c = aperture height, d = spire height, e = aperture breadth, α = apex angle, β = angle between body whorl suture and horizontal surface. Scale bar: 1 mm.

**Figure 3.**
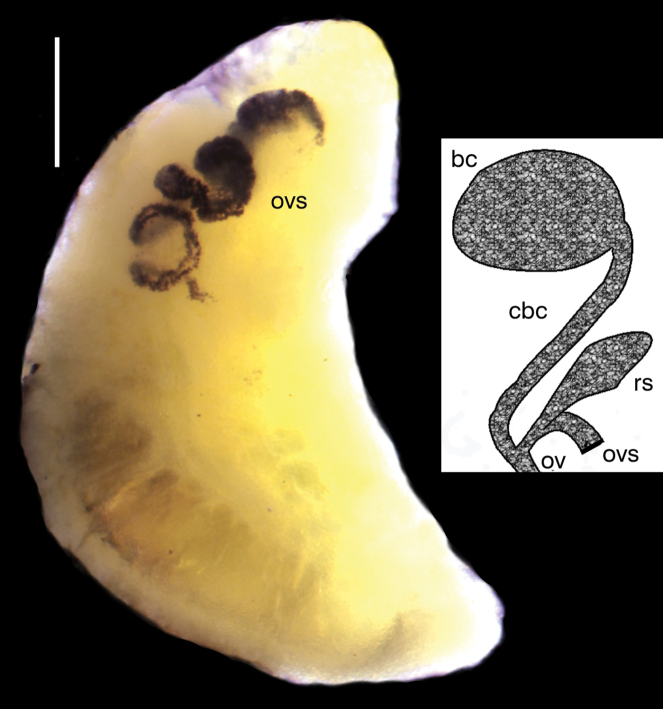
Renal and pallial section of female reproductive organs of *Achaiohydrobia moreana*; drawing not to the scale (bc – bursa copulatrix, cbc – duct of the bursa, ov – oviduct, ovs – spiral of renal oviduct, rs – receptaculum seminis). Scale bar: 200 μm.

**Figure 4.**
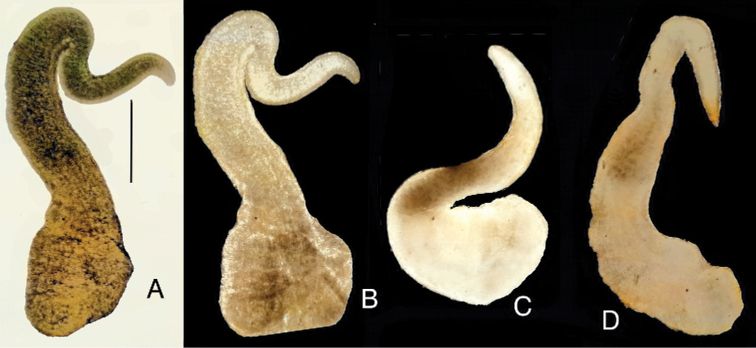
Penis of *Achaiohydrobia moreana*. Scale bar: 200 μm.

The shells (Fig. 2) are broad, ovate-conic with a few flat whorls, rapidly growing and separated by moderately deep suture. Shell measurements are presented in Table 2. Nearly all adult shells have a corroded apex, and most display some injuries, and secondarily healed crevices, perhaps caused by the corrosion by sulphides. Very frequently there are scalariform shells, looking like they were secondarily formed after dissolution of the “normal” shell. The snout and tentacles are uniformly and intensively black pigmented. The female reproductive organs (Fig. 3) have a prominent spiral of black-pigmented renal oviduct. The penis (Fig. 4) has a broad proximal part and long and narrow filament, without any outgrowth. The character states listed briefly above allow the classification to neither known species nor any known genus in the Hydrobiidae.

**Table 2.** Shell measurements of *Achaiohydrobia moreana*; specimen symbols as in Fig. 2; measured variables: see Fig. 2.

	*a*	*b*	*c*	*d*	*e*	α	β
**A – holotype**	**2.25**	**1.29**	**1.06**	**0.63**	**0.82**	**111**	**11**
B – 2A19	2.69	1.34	1.10	0.80	0.91	95	12
C – 2A20	1.68	0.99	0.92	0.38	0.67	98	8
D	1.93	1.02	0.90	0.60	0.63	93	13
E	2.09	1.22	0.96	0.56	0.72	104	15
F	2.45	1.33	1.10	0.69	0.87	109	13
*M*	2.18	1.20	1.01	0.61	0.77	101.67	12.00
*SD*	0.363	0.156	0.091	0.140	0.113	7.474	2.366
*MIN*	1.68	0.99	0.90	0.38	0.63	93.00	8.00
*MAX*	2.69	1.34	1.10	0.80	0.91	111.00	15.00

**Figure 5.**
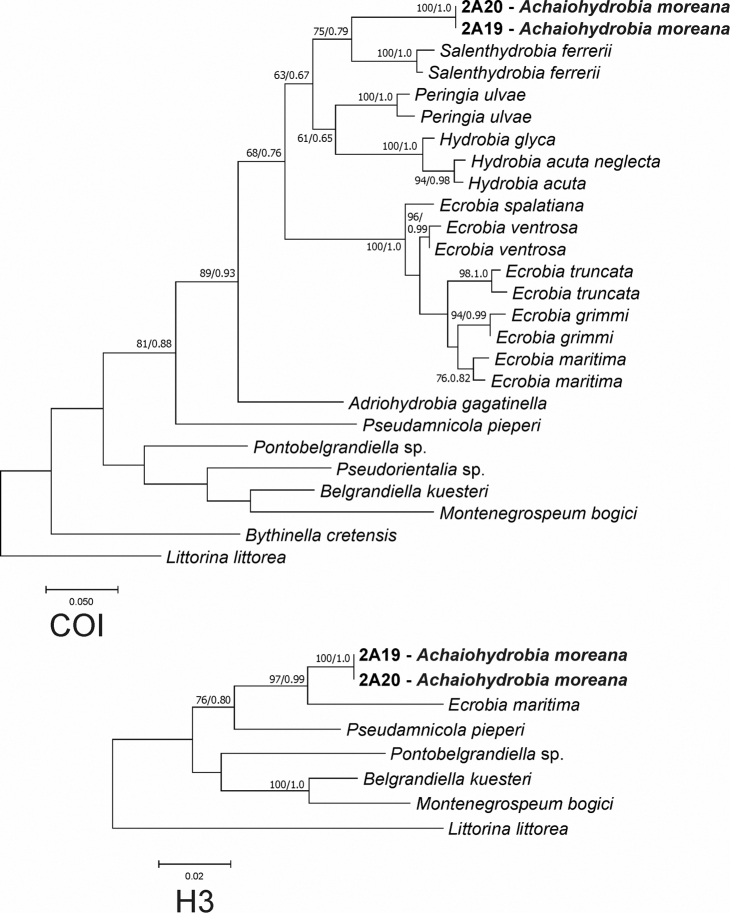
Phylogenetic relationships between the species formerly classified as *Hydrobia*.

We obtained two new sequences of COI (479 bp, GenBank Accession Numbers MW741741-MW741742), and two new sequences of H3 (309 bp, GenBank Accession Numbers MW776415-MW776416). The tests by Xia et al. (2003) for COI and H3 revealed no saturation. In all analyses, the topologies of the resulting phylograms were identical in both the ML and BI. The phylogram based on the cytochrome oxidase (Fig. 5) clearly show the position of this taxon within the “*Hydrobia*” as widely understood, and as a sister taxon (bootstrap support 75%) of *Salenthydrobia ferreri*, with an estimated divergence time of 5.75 ± 0.49 Mya. The pairwise *p*-distances (Table 3) calculated for the taxa shown in the phylogram (Fig. 5) are typical rather for inter-generic level.

**Table 3.** Pairwise genetic *p*-distances calculated for COI within *Hydrobia* s. lato.

***p*-distances between and within genera**					
	* Achaiohydrobia *	* Salenthydrobia *	* Peringia *	* Hydrobia *	* Ecrobia *
* Achaiohydrobia *	*0.000*				
* Salenthydrobia *	0.109	*0.016*			
* Peringia *	0.123	0.121	*0.021*		
* Hydrobia *	0.142	0.131	0.115	*0.028*	
* Ecrobia *	0.167	0.151	0.145	0.150	*0.050*
***p*-distances between *Ecrobia* species**					
	*E. spalatiana*	*E. ventrosa*	*E. truncata*	*E. grimmi*	
*E. spalatiana*					
*E. ventrosa*	0.038				
*E. truncata*	0.064	0.060			
*E. grimmi*	0.066	0.062	0.064		
*E. maritima*	0.057	0.050	0.057	0.040	
***p*-distance between *Hydrobia* species**					
	*H. glyca*	*H. acuta_neglecta*			
*H. glyca*					
*H. acuta_neglecta*	0.037				
*H. acuta*	0.034	0.013			
***p*-distance within *Peringia ulvae*** 0.021					
***p*-distance within *Salenthydrobia ferrerii*** 0.016					

### Family Hydrobiidae Stimpson, 1865

#### Genus Achaiohydrobia

Taxon classificationAnimaliaLittorinimorphaHydrobiidae

Falniowski gen. nov.

B9B4BB40-FB21-58B6-8E13-5C2B5561DFC1

http://zoobank.org/B18334FD-AB44-421A-80FE-568854549087

##### Type species.

*Achaiohydrobia moreana* by monophyly.

##### Diagnosis.

Shell broad, ovate-conic with a few flat whorls, rapidly growing and separated by a moderately deep suture; female reproductive organs with prominent, massive swelling of the spiral of the oviduct; oval bursa copulatrix with the duct longer than the bursa, receptaculum seminis prominent but smaller than the bursa, with the duct slightly distinguishable; penis tapering, widened at the base, without any outgrowths (nonglandular lobes) and without the distal papilla.

##### Derivatio nominis.

The genus name refers to Achaia, one of the ancient names of Greece and the Greek people.

##### Remarks.

The tapering penis with its broad base distinguishes *Achaiohydrobia* from *Hydrobia* and *Peringia*. The lack of any non-glandular outgrowths (lobes) distinguishes it from *Hydrobia*, *Peringia*, and *Ventrosia*. The lack of the distal papilla on the penis distinguishes it from *Salenthydrobia*. The massive swelling of the long spiral renal oviduct differentiates *Achaiohydrobia* from all other genera besides *Hydrobia*. The molecular divergence between *Achaiohydrobia* and the other genera (*p* = 0.109–0.167 for mitochondrial COI) is typical of the genus-level in Hydrobiidae.

#### Achaiohydrobia moreana

Taxon classificationAnimaliaLittorinimorphaHydrobiidae

Hofman & Grego sp. nov.

ABE9365B-1142-5179-A958-42710DE42FA3

http://zoobank.org/165D82EB-2F0A-47DB-BB52-A216E12A99DF

Figure 2

##### Holotype.

GNHM 39589, leg. M. Szarowska and A. Falniowski, 16.07.2009; ethanol-fixed specimen (Fig. 2A), brackish water of Moustos spring, 2 km N of Aghios Andreas, Arkadia, eastern Peloponnese, Greece (37.3845, 22.7444), creeping on the stones and gravel.

##### Paratypes.

Ten paratypes, ethanol-fixed, in the collection of the Department of Malacology of Jagiellonian University. GNHM 39590/20, HNHM Moll.105301/30 wet specimens; NHMW 113630/10 wet specimens; NHMUK 20210006/10; coll. Erőss 10 wet specimens; coll. Grego 32 wet and 36 dry specimens.

##### Diagnosis.

Shell broad, ovate-conic with a few flat whorls, rapidly growing and separated by a moderately deep suture; female reproductive organs with a prominent, massive swelling of the spiral of the oviduct; oval bursa copulatrix with the duct longer than the bursa, receptaculum seminis prominent but smaller than the bursa, with the duct slightly distinguishable; penis tapering, widened at the base, without any outgrowths (no glandular lobes) and without the distal papilla (diagnosis the same as for this monotypic genus).

##### Description.

***Shell*** (Fig. 2) broad, thick-walled, brownish, moderately translucent, up to 2.69 mm high and 1.34 mm broad, ovate-conic with about five flat whorls, growing rapidly and separated by moderately deep suture. Conical spire height about 0.25 of the shell height; body whorl prominent and broad. Aperture narrow, elongate-elliptical; peristome complete and thin, in contact with the wall of the body whorl; umbilicus slit-like or completely covered by the parietal lip. Shell surface smooth, with growth lines hardly visible, often corroded.

***Measurements*** of holotype and sequenced and illustrated shells: Table 2. Shell variability slight (Fig. 2).

**Figure 6.**
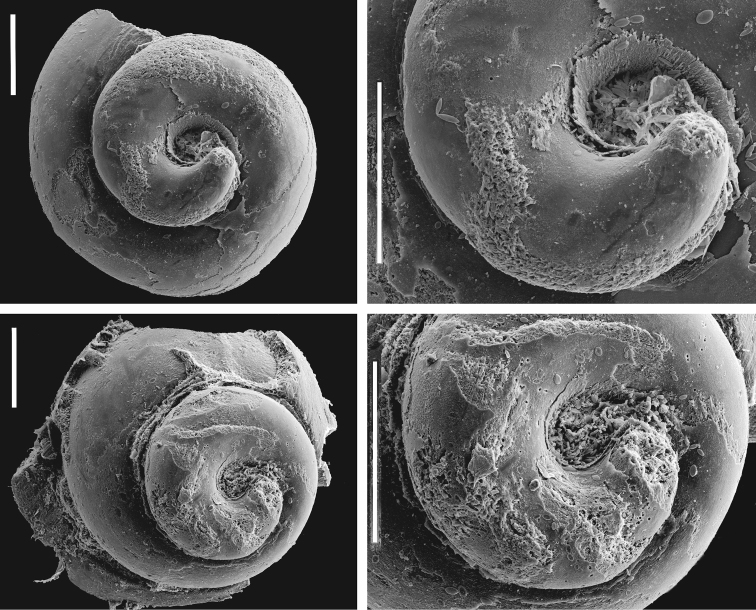
Protoconchs of *Achaiohydrobia moreana*. Scale bar: 200 μm.

***Protoconch*** (Fig. 6) smooth, often corroded.

**Figure 7.**
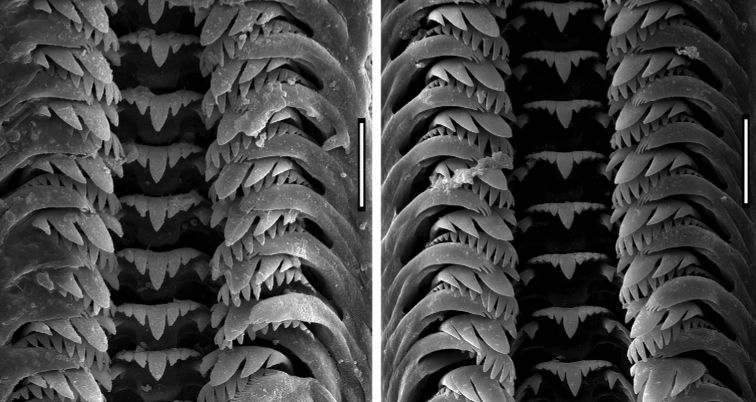
Radulae of *Achaiohydrobia moreana*. Scale bar: 25 μm.

***Radula*** (Fig. 7) typically taenioglossate, with prominent basal cusps and the central cusp on the central toot; central tooth formula:

$\frac{3-1-3}{1-1}$ or $\frac{\left(4)3-1-3(4\right)}{1-1}$ or $\frac{4-1-4}{1-1}$

or or

The lateral tooth with 2 – 1 – 2 prominent and broad cusps, especially the bigger one. Inner marginal tooth with about 11, and outer marginal one with 16–19 cusps.

***Soft parts morphology and anatomy*.** The mantle, snout, and tentacles intensively pigmented uniformly black. The female reproductive organs (Fig. 3) with a prominent, massive swelling of the spiral of the intensively pigmented black renal oviduct. The bursa copulatrix oval, situated dextro-lateral to the style sac, with the duct longer than the bursa, the receptaculum seminis big, but smaller than the bursa, elongated, with the duct slightly distinguishable from the receptaculum. The penis (Fig. 4) tapering, widened at the base, without any nonglandular lobes, no papilla, its proximal section slightly broadened (if at all; see Fig. 4D).

##### Derivatio nominis.

The specific epithet *moreana* refers to Morea, the medieval name of Peloponnese Peninsula.

## Discussion

The radula was the first internal structure considered in the gastropod systematics. In the snails formerly known as *Hydrobia*, whose shells especially lack taxonomically useful characters, the radulae were studied for a long time (Meyer and Möbius 1872; Woodward 1891; Kuhlgatz 1898; Krull 1935; Seifert 1935; Feliksiak 1938; Muus 1963, 1967; Bishop 1976; Falniowski et al. 1977; Bank and Butot 1984; Giusti and Pezzoli 1984; Wilke 2003). However, no constant differences were found to distinguish species, although some not quite stable differences in cusp basal cusp number, etc., were observed. The radula of our *Achaiohydrobia* also bears no unique character states.

The penis morphology of *Achaiohydrobia* is most similar to that of *Adriohydrobia*, but in the newly described genus it is more massive. Differentiating character states are the shape of the bursa copulatrix, its shorter duct and the massive swelling of the oviduct, traits not observable in *Adriohydrobia*. There are detailed data describing the differences in the female reproductive organs (for a summary see Wilke 2003). However, the examination of more numerous specimens (Falniowski 1988) has shown a much variability (also artefactual, physiological, etc.) which gives doubts to the usefulness of these character states for species or genus discriminations (Falniowski 1987, 1989, 1990). The broad base of the penis, listed by Wilke (2003) as one of the apomorphic traits of *Salenthydrobia*, can hardly be recognized as a synapomorphy characterizing all the specimens of our *Achaiohydrobia*. Only three specimens of *Achaiohydrobia* were photographed, and in one of them, no broad base was observed (Fig. 4D).

Brackish-water snails are considered to form isolated populations in suitable habitats, isolated by land, but also by the sea with its full salinity (e.g., Giusti and Pezzoli 1984; Falniowski 1987; Fretter and Graham 1994; Wilke and Davis 2000; Wilke et al. 2000; Wilke 2003). Only *P. ulvae*, capable of living in full sea salinity and with lecithotrophic, although short-living, veliger larva, forms populations less affected by isolation. However, considering all data available (see Fretter and Graham 1994 for details), all “*Hydrobia*” taxa studied so far may be able to survive in open-sea salinity. Anyway, partial isolation promotes allopatric speciation in these gastropods. The type locality of *Achaiohydrobia moreana* is one of the springs fed by the complicated system of poljes and sink-holes in Arcadia (Higgins and Higgins 1996). In the geologic history of the region (e.g., Rögl 1998, 1999) there were many events, such as transgressions of the sea, that must have created conditions that would promote speciation.

The estimated time of divergence between *Achaiohydrobia* and the phylogenetically close *Salenthydrobia* (5.75 ± 0.49 Mya) coincides with 5.33 Mya, which is the time of the Oligocene flooding that ended the Messinian salinity crisis; the estimate is comparable to the divergence time between *Salenthydrobia* and *Peringia* (Wilke 2003) and little older than the two species of *Ecrobia* (Osikowski et al. 2016). An estimated 1.7% divergence per million years is comparable with other estimated times of divergence for Hydrobiidae (Wilke 2003; Osikowski et al. 2016).

The Messinian salinity crisis affected all the Recent basins of the Mediterranean (Krijgsman et al. 1999; McKenzie 1999). The uplift of the northern African and southern Iberian margins, probably due to the roll back of the Tethys oceanic lithosphere delaminating bands of lithospheric mantle from beneath the continental margin (Duggen et al. 2003), blocked the passage between the Atlantic and Mediterranean about 5.96 Mya. This resulted in the regression of the sea and a lowering of the water level by more than 1000 m. In the place of the Recent Mediterranean, there was a desert, crossed by the vast canyons of large rivers, with some water bodies too rich and others too poor in salt; thus, it was impossible for marine organisms to inhabit the area. There were at least 10 sea transgressions in the Mediterranean during the Messinian (Hsü 1983). The region of the Recent Sea of Marmara served as a gateway between the Paratethys and the Mediterranean. Frequent marine introgressions fed water bodies (basins) of a “Lago-Mare” character: large and deep, although with brackish habitats. Apart from brackish water bodies, there were also highly saline ones, but nowhere in the Mediterranean there were normal marine conditions. We should note, however, that such brackish water “Lago-Mare” basins were probably inhabitable for brackish-water snails. Later, 5.33 Mya at boundary between the Miocene from Pliocene, the abrupt catastrophic Pliocene transgression of water from the Atlantic, probably caused by gravity-induced slumping from the western margin of the Gibraltar arch into the Atlantic abyssal plains (Duggen et al. 2003), filled the Mediterranean basin with sea water again. The rapid, drastic changes formed barriers for the fresh- and brackish-water fauna, promoting speciation processes in many animals (e.g., Wilke 2003; Huyse et al. 2004; Falniowski et al. 2007).

## Supplementary Material

XML Treatment for Achaiohydrobia

XML Treatment for Achaiohydrobia moreana

## References

[B1] Bank RA Butot LJM (1984) Some more data on *Hydrobia ventrosa* (Montagu, 1803) and “*Hydrobia*” *stagnorum* (Gmelin, 1791) with remarks on the genus *Semisalsa* Radoman, 1974 (Gastropoda, Prosobranchia, Hydrobioidea). Malakologische Abhandlungen, Staatliches Museum für Tierkunde Dresden 10: 5–15.

[B2] Bank RA Butot LJM Gittenberger E (1979) On the identity of *Helix stagnorum* Gmelin, 1791, and *Turbo ventrosus* Montagu, 1803 (Prosobranchia, Hydrobiidae). Basteria 43: 51–60.

[B3] Bishop MJ (1976) *Hydrobia neglecta* Muus in the British Isles. Journal of Molluscan Studies 42: 319–326. 10.1093/oxfordjournals.mollus.a065340

[B4] Davis GM (1992) Evolution of prosobranch snails transmitting Asian *Schistosoma*: coevolution with *Schistosoma*: a review. Progress in Clinical Parasitology 3: 145–204. https://doi.org/10.1007%2F978-1-4612-2732-8_6 10.1007/978-1-4612-2732-8_6 8420602

[B5] Davis GM McKee M Lopez G (1989) The identity of *Hydrobia truncata* (Gastropoda: Hydrobiidae): Comparative anatomy, molecular genetics, ecology. Proceedings of the Academy of the Natural Sciences of Philadelphia 140: 247–266. https://www.jstor.org/stable/4064963

[B6] Draparnaud J-P-R (1805) Histoire naturelle des mollusques terrestres et fluviatiles de la France. Levrault & Schoell, Paris [2 pp. (Avertissement a sa Majesté l’Impératrice), 2 pp. Rapport, i–viii (Préface) +] 164 pp. [+ pls 1–13, 1 p. Errata.] 10.5962/bhl.title.12856

[B7] Duggen S Hoernie K van den Bogaard P Rüpke L Morgan JP (2003) Deep roots of the Messinian salinity crisis. Nature 422: 602–606. 10.1038/nature01553 12686997

[B8] Edgar RC (2004) MUSCLE: multiple sequence alignment with high accuracy and high throughput. Nucleic Acids Research 32: 1792–1797. 10.1093/nar/gkh340 15034147 PMC390337

[B9] Evagelopoulos A Spyrakos E Koutsoubas D (2009) Phytoplankton and macrofauna in the low salinity ponds of a productive solar saltworks: spatial variability of community structure and its major abiotic determinants. Global NEST Journal 11: 64–72. 10.30955/gnj.000576

[B10] Falniowski A (1986) Pigmentation in *Hydrobia ulvae* (Pennant, 1777) and *H. ventrosa* (Montagu, 1803) (Hydrobiidae, Prosobranchia) from the Polish Baltic. Acta Hydrobiologica 28: 443–449. 10.12657/folmal.028.025

[B11] Falniowski A (1987) Hydrobioidea of Poland (Prosobranchia: Gastropoda). Folia Malacologica 1: 1–122. 10.12657/folmal.001.001

[B12] Falniowski A (1988) Female reproductive organs of *Hydrobia ulvae* (Pennant, 1777) and *H. ventrosa* (Montagu, 1803) from Puck Bay (Southern Baltic Sea) (Gastropoda, Prosobranchia, Hydrobioidea). Malakologische Abhandlungen, Staatliches Museum für Tierkunde Dresden 13: 27–31.

[B13] Falniowski A (1989) A critical review of some characters widely used in the systematics of higher taxa of freshwater prosobranchs (Gastropoda: Prosobranchia), and a proposal of some new, ultrastructural ones. Folia Malacologica 3: 73–94. 10.12657/folmal.003.005

[B14] Falniowski A (1990) Anatomical characters and SEM structure of radula and shell in the species-level taxonomy of freshwater prosobranchs (Mollusca: Gastropoda: Prosobranchia): a comparative usefulness study. Folia Malacologica 4: 53–142. 10.12657/folmal.004.005

[B15] Falniowski A Szarowska M (2002) Comment on the proposed conservation of *Hydrobia* Hartmann, 1821 (Mollusca, Gastropoda) and *Cyclostoma acutum* Draparnaud, 1805 (currently *Hydrobia acuta*) by the replacement of the lectotype of *H. acuta* with a neotype; proposed designation of *Turbo ventrosus* Montagu, 1803 as the type species of *Ventrosia* Radoman, 1977; and proposed emendation of spelling of *Hydrobiina* Mulsant, 1844 (Insecta, Coleoptera) to *Hydrobiusina*, so removing the homonymy with Hydrobiidae Troschel, 1857 (Mollusca). Bulletin of Zoological Nomenclature 59: 128–130.

[B16] Falniowski A Dyduch A Smagowicz K (1977) Molluscs from Puck Bay (Baltic Sea) collected in 1973. Acta Zoologica Cracoviensia 12: 507–531.

[B17] Falniowski A Szarowska M Grzmil P (2007) *Daphniola* Radoman, 1973 (Gastropoda: Hydrobiidae): shell biometry, mtDNA, and the Pliocene flooding. Journal of Natural History 41: 2301–2311. 10.1080/00222930701630733

[B18] Falniowski A Szarowska M Sirbu I Hillebrand A Baciu M (2008) *Heleobia dobrogica* (Grossu & Negrea, 1989) (Gastropoda: Rissooidea: Cochliopidae) and the estimated time of its isolation in a continental analogue of hydrothermal vents. Molluscan Research 28: 165–170.

[B19] Falniowski A Pešić V Glöer P (2014) *Montenegrospeum* Pešić et Glöer, 2013: a representative of Moitessieriidae? Folia Malacologica 22: 263–268. 10.12657/folmal.022.023

[B20] Feliksiak S (1938) Radula von *Hydrobia ulvae* (Pennant) aus der *Littorina*-Zeit. Zoologischer Anzeiger 122: 186–189.

[B21] Fretter V Graham A (1994) British Prosobranch Molluscs. Their Functional Anatomy and Ecology. Revised and Updated Edition. The Ray Society, London, 755 pp.

[B22] Gittenberger E (1991) What about non-adaptive radiation? Biological Journal of the Linnean Society 43: 263–272. 10.1111/j.1095-8312.1991.tb00598.x

[B23] Giusti F Pezzoli E (1984) Notulae malacologicae XXIX. Gli Hydrobiidae salmastri delle acque costiere Italiane: Primi cenni sulla sistematica del gruppo e sui caratteri distintivi delle singole morfospecie. Lavori della Società Italiana di Malacologia 21: 117–148.

[B24] Grego J Hofman S Mumladze L Falniowski A (2017) *Agrafia* Szarowska et Falniowski, 2011 (Caenogastropoda: Hydrobiidae) in the Caucasus. Folia Malacologica 25: 237–247. 10.12657/folmal.025.025

[B25] Grego J Glöer P Rysiewska A Hofman S Falniowski A (2018) A new *Montenegrospeum* species from south Croatia (Mollusca: Gastropoda: Hydrobiidae). Folia Malacologica 26: 25–34. 10.12657/folmal.026.004

[B26] Hall TA (1999) BioEdit: a user-friendly biological sequence alignment editor and analysis program for Windows 95/98/NT. Nucleic Acids Symposium Series 41: 95–98.

[B27] Hartmann JDW (1821) System der Erd- und Flußschnecken der Schweiz. Mit vergleichender Aufzählung aller auch in den benachbarten Ländern, Deutschland, Frankreich und Italien sich vorfindenden Arten. In: Steinmüller JR (Ed.) Neue Alpina.Eine Schrift der Schweizerischen Naturgeschichte, Alpen- und Landwirthschaft gewiedmet, Erster Band. Winterthur, 194–268. [pls 1, 2] http://biodiversitylibrary.org/page/41756566

[B28] Higgins MD Higgins R (1996) A Geological Companion to Greece and the Aegean. Cornell University Press, Ithaca / Duckworth Publishers, London, 240 pp.

[B29] Hsü KJ (1983) The Mediterranean was a Desert. Princeton University Press, Princeton, 216 pp.

[B30] Huyse T Van Houdt J Volckaert FAM (2004) Paleoclimatic history and vicariant speciation in the “sand goby” group (Gobiidae, Teleostei). Molecular Phylogenetics and Evolution 32: 324–336. 10.1016/j.ympev.2003.11.007 15186817

[B31] International Commission on Zoological Nomenclature (2003) Opinion 2034 (Case 3087). *Hydrobia* Harmann, 1821: conserved by replacement of the lectotype of *Cyclostoma acutum* Draparnaud, 1805 (currently *Hydrobia acuta*; Mollusca, Gastropoda) with a neotype; *Ventrosia* Radoman, 1977. Bulletin of Zoological Nomenclature 60: 152–154.

[B32] Kevrekidis T Wilke T Mogias A (2005) When DNA puts ecological works back on the right track: genetic assessment and distribution patterns of mudsnail populations in the Evros Delta lagoons. Archiv für Hydrobiologie 162: 19–35. 10.1127/0003-9136/2005/0162-0019

[B33] Koutsoubas D Arvanitidis C Dounas C Drummond L (2000) Community structure and dynamics of the Molluscan fauna in a Mediterranean lagoon (Gialova lagoon, SW Greece). Belgian Journal of Zoology 130: 131–138.

[B34] Krijgsman W Hilgen FJ Raffi I Sierro FJ Wilson DS (1999) Chronology, causes and progression of the Messinian salinity crisis. Nature 400: 652–655. 10.1038/23231

[B35] Krull H (1935) Anatomische Untersuchungen an einheimischen Prosobranchiern und Beiträge zur Phylogenie der Gastropoden. Zoologische Jahrbücher (Anatomie Ontogenie) 60: 399–464.

[B36] Kuhlgatz T (1898) Untersuchungen über die Fauna der Schwentinemündung mit besonderer Berücksichtigung der Copepoden des Planktons. Wissenschaftliche Meeresuntersuchungen, Kiel, Neue Folge 3: 91–157.

[B37] Kumar S Stecher G Tamura K (2016) MEGA7: Molecular Evolutionary Genetics Analysis version 7.0 for bigger datasets. Molecular Biology and Evolution 33: 1870–1874. 10.1093/molbev/msw054 27004904 PMC8210823

[B38] Küster HC (1852–1853) Die Gattungen *Paludina*, *Hydrocaena* und *Valvata*. In: Abbildungen nach der Natur mit Beschreibungen. Systematisches Conchylien-Cabinet von Martini und Chemnitz, 2. Edition. Nürnberg: Bauer & Raspe. Bd 1, Abt. 21: 1(21) (113): 1–24, pls 1, 2 (1852); (115): 25–56, pls 3–8 (1852); (119): 57–96, pls 9–14.

[B39] Layton KK Martel AL Hebert PD (2014) Patterns of DNA barcode variation in Canadian marine molluscs PLoS ONE 9: E95003. 10.1371/journal.pone.0095003 PMC3990619 24743320

[B40] McKenzie JA (1999) From desert to deluge in the Mediterranean. Nature 400: 613–614. 10.1038/23131

[B41] Meyer HA Möbius K (1872) Fauna der Kie1er Bucht. Bd. 2. Die Prosobranchia und Lamellibranchia, nebst einem Supplement zu den Opisthobranchia. W. Engelmann, Leipzig, 139 pp.

[B42] Miller MA Pfeiffer W Schwartz T (2010) Creating the CIPRES Science Gateway for inference of large phylogenetic trees. In: Proceedings of the Gateway Computing Environments Workshop (GCE), 14 Nov., New Orleans, LA: 1–8. 10.1109/GCE.2010.5676129

[B43] Montagu G (1803) Testacea Britannica, or natural history of British shells, marine, land, and fresh-water, including the most minute: systematically arranged and embellished with figures. Romsey, London, 606 pp. 10.5962/bhl.title.33927

[B44] Muus BJ (1963) Some Danish Hydrobiidae with the description of a new species *Hydrobia neglecta*. Proceedings of Malacological Societies London 35: 131–138. 10.1093/oxfordjournals.mollus.a064910

[B45] Muus BJ (1967) The fauna of Danish estuaries and lagoons. Meddelelser fra Danmarks Fiskeri og Havundersogelser 5: 1–316.

[B46] Nei M Kumar S (2000) Molecular Evolution and Phylogenetics. Oxford University Press, Oxford, 333 pp.

[B47] Osikowski A Hofman S Georgiev D Kalcheva S Falniowski A (2016) Aquatic snails *Ecrobia maritima* (Milaschewitsch, 1916) and *E. ventrosa* (Montagu, 1803) (Caenogastropoda: Hydrobiidae) in the East Mediterranean and Black Sea. Annales Zoologici 66: 477–486. 10.3161/00034541ANZ2016.66.3.012

[B48] Osikowski A Hofman S Rysiewska A Sket B Prevorčnik S Falniowski A (2018) A case of biodiversity overestimation in the Balkan *Belgrandiella* A. J. Wagner, 1927 (Caenogastropoda: Hydrobiidae): molecular divergence not paralleled by high morphological variation. Journal of Natural History 52: 323–344. 10.1080/00222933.2018.1424959

[B49] Paladilhe A (1874) Monographie de nouveau genre *Peringia*, suivie des descriptions d’espčces nouvelles de Paludinées françaises. Annales des Sciences Naturelles, series 6, Zoologie et Paléontologie 1: 1–38.

[B50] Pennant T (1777) British zoology. Vol. IV. Crustacea. Mollusca. Testacea. White, London, 154 pp.

[B51] Radoman P (1973) New classification of fresh and brackish water Prosobranchia from the Balkans and Asia Minor. Posebna Izdanja, Prirodnjacki muzej u Beogradu 32: 1–30.

[B52] Radoman P (1977) Hydrobiidae auf der Balkanhalbinsel und in Kleinasien. Archiv für Molluskenkunde 107: 203–223.

[B53] Radoman P (1983) Hydrobioidea a superfamily of Prosobranchia (Gastropoda). I Systematics. Serbian Academy of Sciences and Arts, Monographs 547, Department of Sciences 57: 1–256.

[B54] Ronquist F Teslenko M van der Mark P Ayres DL Darling A Höhna S Larget B Liu L Suchard MA Huelsenbeck JP (2012) Efficient Bayesian phylogenetic inference and model choice across a large model space. Systematic Biology 61: 539–542. 10.1093/sysbio/sys029 22357727 PMC3329765

[B55] Rueden DT Schindelin J Hiner MC Dezonia BE Walter AE Arena ET Eliceiri KW (2017) ImageJ2: ImageJ for the next generation of scientific image data. BMC Bioinformatics 18: e529. 10.1186/s12859-017-1934-z PMC5708080 29187165

[B56] Rögl F (1998) Palaeogeographic considerations for Mediterranean and Paratethys seaways (Oligocene to Miocene). Annalen des Naturhistorischen Museums in Wien 99A: 279–310.

[B57] Rögl F (1999) Mediterranean and Paratethys. Facts and hypotheses of an Oligocene to Miocene paleogeography (short overview). Geologica Carpathica 50: 339–349.

[B58] Rysiewska A Georgiev D Osikowski A Hofman S Falniowski A (2016) *Pontobelgrandiella* Radoman, 1973 (Caenogastropoda: Hydrobiidae): a recent invader of subterranean waters? Journal of Conchology 42: 193–203.

[B59] Sanderson MJ (2002) Estimating absolute rates of molecular evolution and divergence times: a penalized likelihood approach. Molecular Biology and Evolution 19: 101–109. 10.1093/oxfordjournals.molbev.a003974 11752195

[B60] Sanderson MJ (2003) R8s: inferring absolute rates of molecular evolution, divergence times in the absence of a molecular clock. Bioinformatics 19: 301–302. 10.1093/bioinformatics/19.2.301 12538260

[B61] Schütt H (1980) Zur Kenntnis griechischer Hydrobiiden. Archiv für Molluskenkunde 110: 115–149.

[B62] Seifert TR (1935) Bemerkungen zur Artunterscheidung der deutschen Brackwasser-Hydrobien. Zoologischer Anzeiger 110: 233–239.

[B63] Stamatakis A (2014) RaxML Version 8: a tool for phylogenetic analysis and post-analysis of large phylogenies. Bioinformatics 30: 1312–1313. 10.1093/bioinformatics/btu033 24451623 PMC3998144

[B64] Szarowska M Falniowski A (2014) *Ventrosia maritima* (Milaschewitsch, 1916) and *V. ventrosa* (Montagu, 1803) in Greece: molecular data as a source of information about species ranges within the Hydrobiinae (Caenogastropoda: Truncatelloidea). Folia Malacologica 22: 61–67. 10.12657/folmal.022.006

[B65] Szarowska M Hofman S Osikowski A Falniowski A (2014) Divergence preceding Island formation among Aegean insular populations of the freshwater snail genus *Pseudorientalia* (Caenogastropoda: Truncatelloidea). Zoological Science 31: 680–686. 10.2108/zs140070 25284387

[B66] Szarowska M Osikowski A Hofman S Falniowski A (2016a) *Pseudamnicola* Paulucci, 1878 (Caenogastropoda: Truncatelloidea) from the Aegean Islands: a long or short story? Organisms Diversity and Evolution 16: 121–139. 10.1007/s13127-015-0235-5

[B67] Szarowska M Osikowski A Hofman S Falniowski A (2016b) Do diversity patterns of the spring-inhabiting snail *Bythinella* (Gastropoda, Bythinellidae) on the Aegean Islands reflect geological history? Hydrobiologia 765: 225–243. 10.1007/s10750-015-2415-x

[B68] Tajima F (1993) Simple methods for testing molecular clock hypothesis. Genetics 135: 599–607. 10.1093/genetics/135.2.599 8244016 PMC1205659

[B69] Wilke T (2003) *Salenthydrobia* gen. nov. (Rissooidea: Hydrobiidae): a potential relict of the Messinian salinity crisis. Zoological Journal of the Linnean Society 137: 319–336. 10.1046/j.1096-3642.2003.00049.x

[B70] Wilke T Davis GM (2000) Infraspecific mitochondrial sequence diversity in *Hydrobia ulvae* and *Hydrobia ventrosa* (Hydrobiidae: Rissoacea: Gastropoda): Do their different life histories affect biogeographic patterns and gene flow? Biological Journal of the Linnean Society 70: 89–105. 10.1006/bijl.1999.0388

[B71] Wilke T Falniowski A (2001) The genus *Adriohydrobia* (Hydrobiidae: Gastropoda): polytypic species or polymorphic populations? Journal of Zoological Systematics and Evolutionary Research 39: 227–234. 10.1046/j.1439-0469.2001.00171.x

[B72] Wilke T Pfenninger M (2002) Separating historic events from recurrent processes in cryptic species: phylogeography of mud snails (*Hydrobia* spp.). Molecular Ecology 11: 1439–1451. 10.1046/j.1365-294X.2002.01541.x 12144664

[B73] Wilke T Rolán E Davis GM (2000) The mudsnail genus *Hydrobia* s.s. in the northern Atlantic and western Mediterranean: a phylogenetic hypothesis. Marine Biology 137: 827–833. 10.1007/s002270000407

[B74] Woodward BB (1892) On the radula of *Paludestrina jenkinsi* Smith and that of *P. ventrosa* Mont. Annales and Magazine of Natural History 9: 376–378. 10.1080/00222939208677341

[B75] Vandendorpe J van Baak CGC Stelbrink B Delicado D Albrecht C Wilke T (2019) Historical faunal exchange between the Pontocaspian Basin and North America. Ecology and Evolution 9: 10816–10827. 10.1002/ece3.5602 31632651 PMC6787871

[B76] Xia X (2000) Data analysis in molecular biology and evolution. Kluwer Academic Publishers, Boston, Dordrecht & London, 280 pp.

[B77] Xia X (2013) DAMBE: A comprehensive software package for data analysis in molecular biology and evolution. Molecular Biology and Evolution 30: 1720–1728. 10.1093/molbev/mst064 23564938 PMC3684854

[B78] Xia X Xie Z Salemi M Chen L Wang Y (2003) An index of substitution saturation and its application. Molecular Phylogenetics and Evolution 26: 1–7. 10.1016/S1055-7903(02)00326-3 12470932

